# A way to track governments’ response and people’s mobility changes in response to COVID-19 pandemic

**DOI:** 10.7189/jogh.10.020345

**Published:** 2020-12

**Authors:** Dongshan Zhu, Shiva Raj Mishra, Salim S Virani

**Affiliations:** 1 Centre for Health Management and Policy Research, School of Public Health, Cheeloo College of Medicine, Shandong University, Jinan, China; 2 World Heart Federation, Geneva, Switzerland; 3 Nepal Development Society, Chitwan, Bagmati Province, Nepal; 4 Michael E. DeBakey VA Medical Center and Baylor College of Medicine, Houston, Texas, USA

Social-control measures, medications and a vaccine are key weapons against the pandemic [1]. Before specific medications and vaccines are proven to be effective and can be used worldwide, physical distancing measures are still highly relevant in the fight against COVID-19 [2,3]. Governments around the globe have been taking a wide range of physical distancing measures in response to the COVID-19 outbreak, such as school closure, workplace closure, cancellations of mass gatherings and stay at home orders. However, it’s hard to compare the measures implemented in different countries directly. First, a same measure might be implemented in different countries with different degree and intensity. Also, citizens’ acceptability of the measure differs across countries.

## THE GOVERNMENTS’ STRINGENCY INDEX AND COMMUNITY MOBILITY REPORTS

The University of Oxford has been publishing The Oxford COVID-19 Government Response Tracker (OxCGRT) since April 2020 [4,5]. For each country, nine sub-indicators (school closing, workplace closing, cancel public events, restrictions on gatherings, close public transport, stay at home requirements, restrictions on internal movement, international travel controls, and public information campaigns) are synthesized into a common Stringency Index (SI) (a value between 0 and 100) that reflects the overall stringency of a government’s response to COVID-19 pandemic on a daily basis. Also, on April 3, 2020, Google published the “COVID-19 Community Mobility Reports” which allows assessment of the mobility patterns in response to the recommended policies from February 15 [6]. Deidentified and population level aggregated reports for mobility patterns (decreased or increased) in visiting six categories of places were reported: workplaces, retail and recreational venues, groceries and pharmacies, parks, transit stations, and places of residence.

## A WAY TO TRACK GOVERNMENTS’ RESPONSE AND PEOPLE’S MOBILITY CHANGES

**Figure 1:**
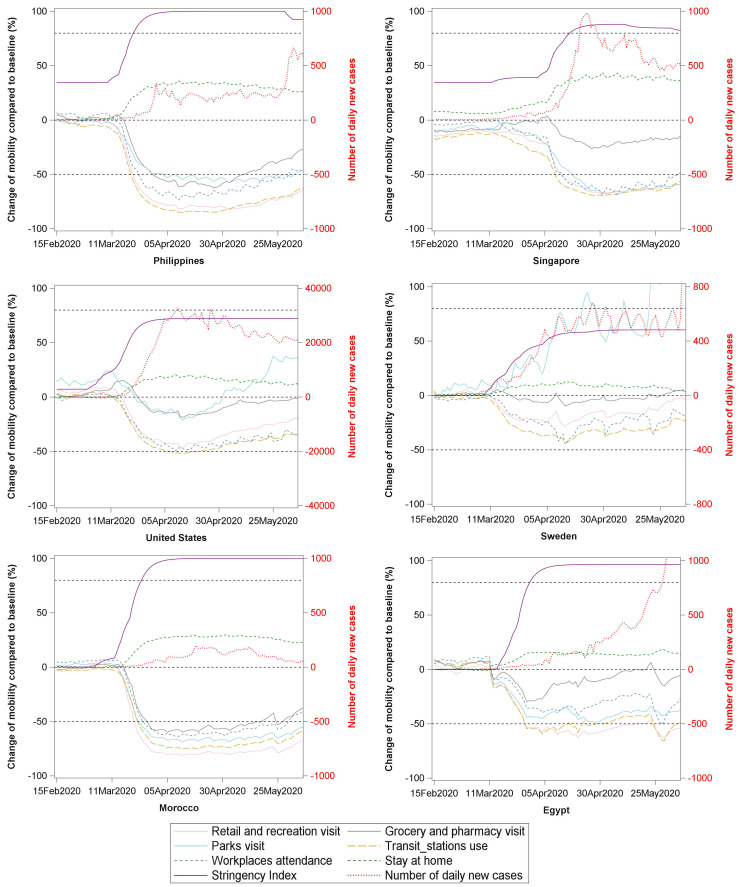
The trajectory of Stringency Index (SI), daily new cases, and changes of six categories of community mobility. An exponentially weighted moving average method with parameter 0.3 was used to smooth time series of SI and number of daily new cases, and a base-10 log scale was used for the y-axis for daily new cases).

Trajectory of SI reflects the speed and strength of a country's response, while trajectories of mobility changes reflect citizens’ degree of acceptance and degree of adherence to the governments’ recommendations or requirements. We hypothesized that combining the SI and mobility report would be an effective way to track both the governments’ response and citizens’ behaviour changes during the COVID-19 pandemic. Six countries’ (from Asia, America, Europe and Africa, respectively) trajectory of SI, number of daily new cases, and changes in six categories of community mobility were plotted for visualization (**Figure 1**).

After March 11 (the day WHO declared a pandemic spread of COVID-19), most countries upgraded their response level, reflected by an increasing SI. The Philippines, Singapore, Morocco and Egypt upgraded SI to a high level (SI>80), while the USA and Sweden raised SI to a moderate level of less than 70. Under different SI, citizens’ mobility changes differed in these countries. The extent of divergences between SI and mobility patterns’ changes reflect both the stringency level of a government’s response and the degree of compliance from citizens to these control measures. We identified several patterns of SI and compliance that are linked to the spread of COVID-19 as follows.

## PATTERNS OF SI AND CITIZENS’ COMPLIANCE ARE LINKED TO THE SPREAD OF COVID-19

Both the Philippines and Singapore initiated a response from January and maintained a low to moderate SI for a while. After March 11, the Philippines government upgraded SI to a rather high level (SI>90). Under this SI, outside visits to the five places, especially the transit station use and retail/recreation visits declined by over 50%. The daily incidence of cases first peaked at around April and maintained a low spread level until the end of May. Intriguingly, daily activities (eg, groceries/pharmacies visit) started to resume from May, while daily incidence had no obvious change until 25 May. This may indicate a time lag between citizens’ mobility changes and the rise in the number of COVID-19 cases. Along with the rebound of COVID-19 spread in migrant workers [7], Singapore raised its SI to a high level (SI>80), coinciding with the distinct decline of outside visits, and the residential stay increased by around 40%. The SI was kept high in Singapore to fight the secondary wave of infection.

**Figure:**
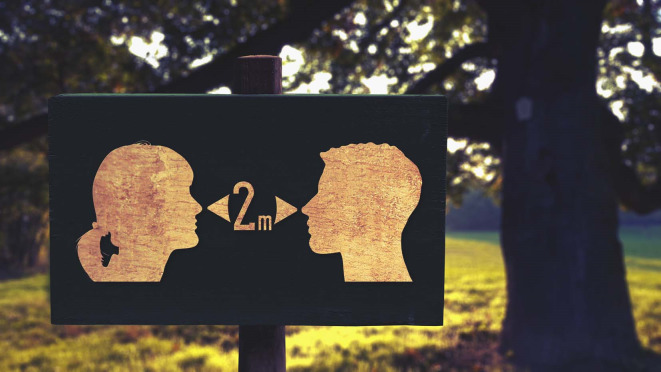
Photo: A sign in the park to remind people to keep distance (from a free image website https://pixabay.com/).

The USA and Sweden all upgraded their SI to a moderate level of less than 70. In the USA, all outside visits decreased less than 50%, and the park visit boomed again from May. The daily incidence has maintained a high plateau from April to date. The Swedish government has maintained a moderate SI level up to now. Citizens’ outside visits declined by less than 30% and the park visit even increased by 50% from April. The daily incidence had a big fluctuation and the spread increased sharply after June. Given the high mortality rate (especially for the elderly) in the USA and Sweden, governments may need to do more [8].

A high SI level does not always mean high compliance from citizens. Among the African countries, both Morocco and Egypt had a SI level greater than 80, but the decline in mobility was much lower in Egypt than Morocco, which may partly explain the sharp rise of COVID-19 in Egypt (**Figure 1**).

It is worth mentioning that a common mobility change in Singapore, the USA, Sweden and Egypt was that a relatively small decline in grocery/pharmacy visits (the grey line in Figure). This may reflect people with symptoms going to the pharmacies to purchase medications for symptom control, without seeking a health service at the first onset of symptoms. Delays in seeking health services can increase the chance of spreading the infection to others.

## CONCLUSIONS

Combining the SI and mobility report is a good way to track both governments’ stringency of response and citizens’ compliance to the measures recommended during the COVID-19 pandemic. It also provides an opportunity to compare the physical measures implemented in different countries and their effectiveness on curbing COVID-19 spread. In addition, some countries started to relax COVID-19 physical distancing measures from May [9]. SI and Community Mobility Report may help policymakers and public health professionals to monitor the impact of policy shift (from stringency to relax) on citizens’ mobility and on the epidemic of COVID-19. These in turn are important to mitigate community spread of COVID-19 infection.

## References

[1] Monto AS Vaccines and antiviral drugs in pandemic preparedness. Emerg Infect Dis. 2006;12:55-60. 10.3201/eid1201.051068 16494718 PMC3291404

[2] Cowling BJ Ali ST Ng TWY Tsang TK Li JCM Fong MW Impact assessment of non-pharmaceutical interventions against coronavirus disease 2019 and influenza in Hong Kong: an observational study. Lancet Public Health. 2020;5:e279-88. 10.1016/S2468-2667(20)30090-6 32311320 PMC7164922

[3] Lai S Ruktanonchai NW Zhou L Prosper O Luo W Floyd JR Effect of non-pharmaceutical interventions to contain COVID-19 in China. Nature. 2020. Online ahead of print. 10.1038/s41586-020-2293-x 32365354 PMC7116778

[4] Blavatnik School of Government UoO. Oxford COVID-19 Government Response Tracker. 2020. Available: https://www.bsg.ox.ac.uk/research/research-projects/coronavirus-government-response-tracker. Accessed: 12 June 2020.

[5] Hale. T, Angrist. N, Kira. B, Petherick. A, Phillips. T, Webster. S. “Variation in Government Responses to COVID-19” Version 5.0. Blavatnik School of Government Working Paper. Available: https://www.bsg.ox.ac.uk/research/publications/variation-government-responses-covid-19. Accessed: 12 June 2020.

[6] Google Inc. COVID-19 Community Mobility Reports. 2020. Available: https://www.google.com/covid19/mobility/index.html?hl=en. Accessed: 12 June 2020.

[7] Hargreaves S Rustage K Nellums LB McAlpine A Pocock N Devakumar D Occupational health outcomes among international migrant workers: a systematic review and meta-analysis. Lancet Glob Health. 2019;7:e872-82. 10.1016/S2214-109X(19)30204-9 31122905 PMC6565984

[8] Giesecke J The invisible pandemic. Lancet. 2020;395:e98. 10.1016/S0140-6736(20)31035-7 32539940 PMC7200128

[9] Mahase E Covid-19: South Korea relaxes social distancing after the number of new cases drops below 10 a day. BMJ. 2020;369:m1842. 10.1136/bmj.m1842 32371381

